# English Wit

**Published:** 1889-07

**Authors:** 


### ENGLISH WIT.

A WRITER in one of our English contemporaries comments rather sarcastically upon an American editor who signed himself as "Editor and Bus. Manager," suggesting that it "smacked of that carryall commonly seen upon the streets." There is an old saying that "brevity is the soul of wit," and in the above signature there is "more truth than poetry," for those who have had the pleasure of experiencing the labors of a position indicated by ye editor's title know full well that he is not only a "common conveyance," in many instances, but a "free hack!" at that.

